# Experiences of general practice care for self-harm: a qualitative study of young people’s perspectives

**DOI:** 10.3399/BJGP.2021.0091

**Published:** 2021-08-03

**Authors:** Faraz Mughal, Lisa Dikomitis, Opeyemi O Babatunde, Carolyn A Chew-Graham

**Affiliations:** School of Medicine, Keele University, Keele; affiliate, NIHR Greater Manchester Patient Safety Translational Research Centre, Keele University, Keele; honorary clinical research fellow, Unit of Academic Primary Care, University of Warwick, Coventry.; School of Medicine, Keele University, Keele.; School of Medicine, Keele University, Keele.; School of Medicine, Keele University, Keele; honorary professor of primary care mental health, Midlands Partnership NHS Foundation Trust, Stafford.

**Keywords:** family medicine, help-seeking behaviour, primary healthcare, qualitative research, self-injurious behaviour, youth

## Abstract

**Background:**

Self-harm is a growing concern and rates of self-harm in young people (aged 12–25 years) presenting to general practice are rising. There is, however, little evidence about young people’s experiences of GP care and on accessing general practice.

**Aim:**

To explore the help-seeking behaviours, experiences of GP care, and access to general practice of young people who self-harm.

**Design and setting:**

In this qualitative study, semi-structured interviews were conducted with young people aged 16–25 years from England with previous self-harm behaviour.

**Method:**

Interviews with 13 young people took place between April and November 2019. Young people were recruited from the community, third-sector organisations, and Twitter. Data were analysed using reflexive thematic analysis with principles of constant comparison. A patient and public involvement advisory group informed recruitment strategies and supported interpretation of findings.

**Results:**

Young people described the avenues of help-seeking they employ and reflected on the mixed experiences of seeing GPs that can influence future help-seeking. Preconceptions and a lack of knowledge about accessing general practice were found to be barriers to help-seeking. GPs who attempt to understand the young person and establish relationship-based care can facilitate young people accessing general practice for self-harm.

**Conclusion:**

It is important young people are aware of how to access general practice and that GPs listen, understand, and proactively follow-up young people who self-harm. Supporting young people with self-harm behaviour requires continuity of care.

## INTRODUCTION

Self-harm in young people is a national public health concern.^1^ Defined as self-poisoning or self-injury regardless of intent, self-harm is the strongest risk factor for suicide, increasing suicide risk by 50 times.^2^^–^3 In young people, self-harm is thought to be influenced by biopsychosocial factors, and is associated with depression, anxiety, future self-harm episodes, and poorer educational and employment outcomes.^4^^–^5

In young people (aged 10–24 years) there is a 26% lifetime prevalence of self-harm, with self-cutting the most prevalent method in the community.^6^^–^7 In young people who have died by suicide, over 50% had a past history of self-harm.^8^ Episodes of self-harm in young people presenting to general practice have increased, and young people who self-harm (aged 16–24 years) see GPs the most in the NHS.^9^^–^12 A fear of negative reactions has been identified as a barrier to accessing services for young people who self-harm, and only a few facilitators have been identified for help-seeking.^13^

A National Institute for Health and Care Excellence (NICE) self-harm guideline research recommendation is that rigorous qualitative research should explore user experiences of services.^14^ GPs report a positive attitude to providing frontline support for young people who self-harm; however, there is little published literature on young people’s experiences of, and access to, care in general practice for self-harm.^15^ The aim of this study was to explore the help-seeking behaviours, experiences of GP care, and access to general practice of young people who self-harm.

## METHOD

This study adopted a qualitative methodology using semi-structured interviews that enabled in-depth exploration of young people’s experiences and perspectives.^16^ This study was informed by constructionist epistemology and a critical realist theoretical stance, and acknowledged that individuals have their own subjective insights dependent on their life experiences.^17^^–^18 This study is reported in accordance with the Standards for Reporting Qualitative Research.^19^

A patient and public involvement advisory group informed this study through revising the interview topic guide, designing recruitment strategies, and interpreting findings.

### Setting and participants

This study was based in England. Young people aged 16–25 years, regardless of type of self-harm were eligible to participate.

### Recruitment

Participants were recruited from the community, Twitter, and self-harm third-sector organisations. The recruitment poster was displayed around some universities in the North of England and the Midlands, local council libraries, and sixth-form colleges. A Twitter recruitment message was written with the patient advisory group and posted on the lead author’s personal account. Eight national self-harm third-sector organisations were contacted by email to ask if they would share the recruitment poster within their organisations. Recruiting purposively, to aim for maximum variation, was attempted but this proved challenging, and thus a national convenience sample was obtained. Interested eligible participants were emailed an invitation letter, study information sheet, and consent form.

**Table:** How this fits in

Young people who self-harm present to GPs in the NHS, but their perceptions of care remain largely unexplored. This qualitative study indicated that young people sought help from a variety of services, including non-statutory services and NHS services. Young people described mixed experiences of consulting GPs, which can influence help-seeking from general practice. A relationship with one GP who listens, appears to understand, and offers proactive follow-up is an important facilitator for young people who access general practice for self-harm.

### Data collection

One author (a GP researcher with expertise in self-harm in primary care) conducted all interviews from April to November 2019. Interviews were digitally recorded, transcribed verbatim by the same author or a professional transcription company, and anonymised.

Interviews were semi-structured to adequately explore and be flexible to the narratives of young people during interviews. Semi-structured interviews have previously been used with young people who self-harm.^20^ Interviews were carried out face-to-face or by telephone. A topic guide developed from the literature, research team discussion, and patient and public involvement advisory group input explored reasons for young people’s self-harm, experiences of GP care, and access to general practice care for self-harm. It was iteratively refined as data collection and analysis matured in parallel.

Consent was confirmed at the start of interviews, and participants were free to withdraw from participating at any time. A study risk protocol was established in case distress was identified in participants during the study process. All participants received a ‘Staying Safe Sheet’ that listed support services for self-harm at the beginning of interviews. Face-to-face interviews were held in private meeting rooms at Keele or Birmingham Universities, and the option of a telephone interview was given.

All participants were offered a 10 GBP Amazon voucher on completion of interview. Data collection stopped when data saturation (no new data were emerging) was felt to be reached.^21^

### Data analysis

Interview data were analysed using reflexive thematic analysis applying principles of constant comparison, compatible with a critical realist stance.^22^^–^23 Analysis was flexible and recursive, moving between stages, and each transcript was coded by the author who conducted the interviews. All transcripts were independently coded by at least two authors. Codes were compared across transcripts, sorted into wider categories, and recorded in an analysis table to support the generation of candidate themes. Higher-level recurring themes were agreed on by all authors. Findings were presented to the patient and public involvement advisory group.

### Reflexivity

The interviewer made field notes after each interview that supported topic guide iteration, the analysis process, and researcher reflexivity. At study meetings, researchers considered how their backgrounds influenced interpretation of the data, and their understanding of findings. The research team members have different professional backgrounds: social science, anthropology, general practice, health services research, and evidence synthesis. This, and the input of the patient and public involvement advisory group into the interpretation of findings, increases the breadth and depth of analysis, and thus the trustworthiness of findings.^24^

## RESULTS

In total, 13 interviews with young people who self-harmed were conducted. Interviews lasted between 25 and 49 min. Nine interviews were face-to-face, and four were by telephone. Participant demographic characteristics are detailed in Table 1. The age of participants ranged from 19–25 years, and participants were from the Midlands and South East England. At the time of interview 12 participants were in higher education (*n* = 5 undergraduate, *n* = 7 postgraduate), and one in further education. Narratives of young people were attained from before, and within, education settings. The risk protocol was not activated during the study.

**Table 1.** Participant demographic characteristics

**Gender**	**Age, years**	**Education status**	**Self-identified ethnicity**
Female	24	PG	Not disclosed
Female	24	PG	White British
Female	21	UG	White British
Female	23	PG	Mixed
Female	22	PG	White British
Female	23	UG	Mixed
Female	25	PG	White British
Female	25	PG	Asian British
Transgender male	21	In college	White British
Female	19	UG	White American
Female	22	PG	White British
Female	19	UG	Mixed
Female	19	UG	White British

*PG = postgraduate. UG = undergraduate.*

The three themes generated are: help-seeking avenues, barriers to seeking help from general practice, and facilitators to accessing general practice care. Themes are supported by illustrative quotes. Unique identifiers for young people include their pseudonym and age.

### Help-seeking avenues

Young people who self-harm described different avenues of help-seeking: role of significant others, non-statutory services, and NHS services.

#### Role of significant others in supporting help-seeking

Participants described how parents and friends enabled them to either seek help or had sought help on their behalf:

*‘In middle school … it was brought to my parents’ attention that I had been cutting myself, and then they took me to see a therapist.’*

(Hannah, 19 years)

*‘But my friends kind of caught on, I didn’t tell them, but they know me well enough to realise what was going on so when they called the services, people were trying to arrange a mental health act assessment.’*

(Bethany, 24 years)

Some participants reported how their partners had influenced their help-seeking behaviour and supported their candidacy (one’s eligibility for medical intervention jointly negotiated between individuals and health services) for care,^25^^–^26 such as:

*‘I really felt pressured* [from her partner] *… to go and seek help, but I myself did not want to do it, but then eventually I did go, and I hmmm obviously didn’t really like doing it first, because I don’t even for physical problems, I don’t go to the GP, I am like, just leave it … do things on my own … and it was my first time I probably said it out loud that I was self-harming.’*

(Lucy, 24 years)

Other participants described how other people had hindered their ability to seek help for self-harm:

*‘And as I got older, I became responsible for caring for my sister. She was very, very, very, very ill for a really long time and I think when I first sought help for it all, was when she had a dissociative identity disorder and, er, when I first sought help for my own self-harm was when I’d taken her in for hers.’*

(Divya, 23 years)

Participants reflected on how parents, partners, and friends could act as enablers for help-seeking and support participant candidacy, whereas others described how other people could hinder their efforts to seek help.

#### Non-statutory services

Participants described seeking help for self-harm from higher education services, third-sector organisations, the internet, and private services:

*‘Last year when that did happen, I made a counselling* [university service] *appointment, I think I went to one or two and then I just picked myself back up.’*

(Hannah, 19 years)

*‘Getting to a point where I was contacting Samaritans like every single day, erm, and getting fed up and reaching the point where like I wouldn’t seek help and you know; I wouldn’t be alive kind of thing.’*

(Ian, 21 years)

Participants also described seeking help and support from online resources:

*‘I looked at a few online resources, so pamphlets … and I think that was quite helpful to relax me about the situation I was in and what support systems are available out there, so I don’t feel alone; I think it just helped me be nudged in the right direction to at least try.’*

(Jemima, 25 years)

Ian detailed that he had sought private psychological therapy following dissatisfaction with statutory services:

*‘I had a further six assessments with them* [community mental health team] *that came to nothing and I reached a point where I was just like you know … I need to do something, like this is becoming a critical stage and so I was like … I need private therapy, I need something to keep me alive.’*

(Ian, 21 years)

Young people described barriers to help-seeking from non-statutory services. Marie shared her experience of seeing up to eight counsellors, but she explained she needed to be ready to seek help for self-harm, highlighting a challenge young people may face when struggling with self-harm:

*‘I don’t think I was quite there in my mind that I was as worse as I was or bad as I was … I know last year for example I was in not a very good place at all and I now know that even though I don’t like it, necessarily talking to counsellors … it will help me in the long run.’*

(Marie, 19 years)

#### NHS services

Some participants described experiences of seeking help from primary care services:

*‘And the first person I spoke to, was the pharmacist … he was totally calm about it … but it was the changing point in my life that I actually realised that it’s not something to be ashamed of.’*

(Kate, 22 years)

Participants stated how they sought help for self-harm through the NHS community-based Improving Access to Psychological Therapies service. Many participants shared negative experiences:

*‘I think the first one was erm, there was a CBT* [cognitive behavioural therapy] *experiment afterwards, these are in like a year break of each other but erm, the CBT person, they wanted to do it over the phone which I found more difficult to begin with and then they were half an hour late for the appointment on the phone so I found that like “okay, you’re not going to turn up to a phone appointment on time then I don’t think that this would work”.’*

(Gemma, 25 years)

As Gemma highlights, psychological therapies over the telephone does not suit some young people. Participants also described varied experiences of seeking help through NHS mental health services:

*‘The first time I spoke to someone about it, it was honestly the most useless … first of all they just told me I was being attention seeking* [Child and Adolescent Mental Health Services counsellor] *so I just kind of, yeah … it took me a while to look for help again … she wasn’t really listening to what I was saying and as she was just finishing the sentences for me.’*

(Emily, 23 years)

A separate experience was shared by Emily that was in contradiction to her first experience with mental health services:

*‘… they* [mental health access team] *did actually like em, ‘cause it was the first time that kind of, that I actually thought that I was being listened to and like, they were trying to actually figure out ways to help me rather than just completing my thoughts.’*

(Emily, 23 years)

Some participants felt frustrated when they were deemed not eligible for NHS psychological therapy services after being referred by clinicians as they struggled with self-harm:

*‘I’ve been referred to psychotherapy ‘cause of my diagnosis* [diagnosis not disclosed] *and they’ve gone, “we can do eight sessions of CBT but we don’t think it’s going to achieve anything and you’re still hurting yourself and it’s against our policy to do that”.’*

(Divya, 23 years)

### Barriers to seeking help from general practice

Young people reflected on their experiences consulting GPs for self-harm. They described what influenced future help-seeking, and how preconceptions of GP care, knowledge on self-harm and of accessing general practice, and fear of consulting GPs were barriers to accessing general practice care.

#### Expectations not met

Some participants vividly described experiences of feeling that a GP did not fully explore their problem and seemed to rely on prescribing as a management option:

*‘But he sort of went “okay we’ll just put you on anti-depressants and see you every two weeks and let’s see what does, see if your mood increases, if anything happens, if you stop self-harming, if things decrease” … I ended up maxing out on the amount you can get with anti-depressants within like six months, and they weren’t sitting well with me.’*

(Gemma, 25 years)

Some accounts revealed tensions in perspectives of experience of previous consultations:

*‘It was … hmphh* [small sigh] *… it was pretty positive, I mean he was, he was understanding, very non-judgemental, warm, I felt comfortable telling him everything … one thing that did not feel quite right was the way he responded … like I told him I don’t know … “I have a sore throat”.’*

(Lucy, 24 years)

Lucy suggested that although she found the GP to be considerate and this supported her self-harm disclosure, she thought he responded casually to her disclosure of self-harm.

#### Preconceptions and fears

Young people held preconceived views of GPs’ care for self-harm, which acted as a barrier and hindered them seeking support for self-harm through general practice:

*‘From what I’ve noticed from others and my own experiences is that they don’t really get good experiences straight away, and then like I said, it took me such a long time before I actually tried again.’*

(Emily, 23 years)

Young people vividly shared fears of being admitted to hospital, loss of confidentiality, and stigma as barriers for accessing support from general practice:

*‘I thought they’d hospitalise me immediately. I thought they’d panic and push me away as if, “no, you’ve gotta — you know, you’ve got to go into an inpatient unit, and we’ve got to inform your family, and you’ve got to quit your course”.’*

(Kate, 22 years)

*‘Erm, it was really, really difficult erm, it’s hard enough trying to get an appointment these days, erm, and it’s when they ask you on the phone, “can I ask you like why you need this appointment”, I just lied and just said, you know, I feel ill … I felt a lot of shame in it.’*

(Ian, 21 years)

As highlighted by Ian, a fear of stigma around self-harm was found to be a barrier to accessing care.

#### Experiences of consulting GPs influence help-seeking

Young people reflected on the importance of GPs’ responses to self-harm when discussing self-harm in the consultation and provided insights into the impact these had on help-seeking, highlighting the potential consequences of a negatively perceived GP consultation.

Some participants described that their experience of seeing GPs for self-harm recursively affected their decision to seek help for self-harm in the future:

*‘Err I dunno it was easy to get a doctor’s appointment but the help I got it wasn’t any help as it then put me back another three years until I got help again … like they printed out a form about manic depression and generalised anxiety disorder and then that was it and that’s all I got … nothing and then I never bothered until another three years later.’*

(Catherine, 21 years)

Other participants, however, reflected on how positive experiences of consulting a GP had resulted in them seeking help from GPs in the future:

*‘I had a really great experience, being able to share that with someone new, also my comfort zone and he* [GP] *just became my support so if I ever had to have an appointment, I just went to him for continuity.’*

(Jemima, 25 years)

Some participants shared that if they felt self-harm was dismissed by GPs after it was mentioned in the consultation, their future help-seeking behaviour changed as a result:

*‘I left the conversation feeling perhaps I was assigning more importance to this that it requires … because I said “if the GP is not too concerned, I shouldn’t be” … I felt I needed to tell him … that I’m actually overdosing on them* [prescribed antidepressants] *… I did tell him, and once again I didn’t get any reaction … so I decided to stop my medication without telling him and I never attended another appointment with him … I’ve never been to see the GP since and it’s been six months.’*

(Lucy, 24 years)

#### Lack of knowledge on self-harm and accessing support

Participants also described how a lack of knowledge about self-harm and its risks, and accessing care in general practice was a barrier to seeking support from general practice:

*‘There’s sort of a lack of knowledge around the healthcare system works and booking GP appointments is scary … also it’s sort of around knowledge where I didn’t necessarily realise that me self-harming was wrong or was a sign that I will* [do so] *for a very long time.’*

(Divya, 23 years)

**Figure 1.**
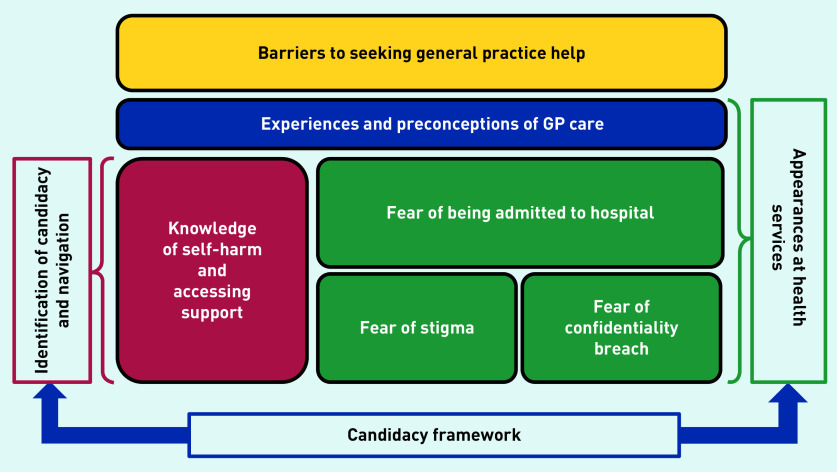
***Diagram illustrating barriers for young people seeking help from general practice.****25^,^^26^*

These barriers are mapped to a candidacy framework and presented in Figure 1.

### Facilitators to accessing general practice care

#### Listening and acting

Participants described positive consultation experiences when GPs were proactive in assessing and managing their self-harm:

*‘Yeah, yeah, yeah it was erm, yeah, my GP here is very helpful, he gives me different, he gives me options and then explains to me which erm, how each go, which things that would be best for me.’*

(Emily, 23 years)

Some participants shared experiences of feeling that GPs were active listeners and non-judgemental, and involved them in shared decision making:

*‘His patience and lack of judgement was amazing, just to listen to my experiences of what happens for emotionally when I’m self-harming, erm, it was incredible.’*

(Kate, 22 years)

*‘He didn’t over-react … he was really good in the way he handled things … the way he felt comfortable to talk to me about it made me comfortable, even though I didn’t feel anything at that time, I didn’t feel as though I was being judged … I was on citalopram and he discussed in detail what the side effects were, what would happen, what the benefit of sertraline were and he said, if you need me to speak to your therapist, I will … he was amenable to helping me with my self-harm.’*

(Jemima, 25 years)

#### Being understood

Young people described wanting to be understood by GPs and be treated as an individual:

*‘It just feels with the GPs, very erm like almost like they are reading from a script with it … as opposed to talking with you about it.’*

(Marie, 19 years)

*‘He could have found out more, asked to find out more and then talked to me more, or at least talked to me about maybe what I wanted to do.’*

(Hannah, 19 years)

Participants reflected that they want GPs to personalise their care and support to them, which may facilitate help-seeking in young people, and reduce self-harm behaviour.

#### Relationship-based care

An important facilitator identified was an ongoing relationship and continuity with GPs for self-harm care:

*‘Definitely a key part is the rapport between the GP and the err … so definitely continuity, relational continuity definitely plays a role in that, especially I would have loved to have a GP I’ve known for a couple of years, perhaps I could have prevented this whole thing from happening.’*

(Lucy, 24 years)

Some participants also suggested that longer GP consultations would support young people accessing general practice self-harm care, and this can support relationship-based care:

*‘Continuity and a good frequency of GP appointments is really helpful. You don’t build up a rapport in one appointment, it’s ten minutes, and some place it’s five minutes, you need time to do that, quite often they’ll book double appointments knowing that I’ve only got one problem.’*

(Kate, 22 years)

#### Shorter waiting times

Participants described that shorter waiting times to see GPs would facilitate access to care for young people with self-harm behaviour:

*‘When you self-harm you like you don’t wanna wait two weeks, you need to see them there or then, or not at all … like it’s very instant, so like if you’re gonna self-harm or have self-harmed, there is no point seeing them in two weeks.’*

(Catherine, 21 years)

## DISCUSSION

### Summary

To the authors’ knowledge, this is one of the first studies to explore young people’s experiences of care and their views on access to care in general practice for self-harm. Young people described avenues of help-seeking encompassing significant others, non-statutory services, and NHS services. Young people reflected on poor GP experiences, and how these influenced future help-seeking. Prior views, a lack of knowledge, and fear were further barriers identified for help-seeking. GPs listening, taking action, showing understanding, and providing relationship-based care, as well as shorter GP waiting times were facilitators to accessing general practice care.

### Strengths and limitations

This study provides new and rich insights in a general practice context about young people who self-harm. The analysis approach used allowed for a fluid analytical process ensuring the full richness of the data were explored in generating themes close to the original data.^22^^–^23 The researcher shared his professional background with participants early on and to build trust and rapport; however, it is acknowledged that doing so may influence the content of participants’ narratives.^27^^–^29 The involvement of the patient and public involvement advisory group in the interpretation of findings allowed the inclusion of lay perspectives, thereby improving the relevance and validity of findings.

Limitations include the possibility of selection bias, as participants who were willing to be interviewed may hold different views from those who were not. Interviews were conducted at one time-point in the young person’s journey of self-harm care. It is therefore not possible to understand how experiences and perspectives progressed over time. Despite efforts to recruit a diverse sample of young people, a convenience sample may not be representative of all young people who self-harm; young people in this study were nearly all female and in further or higher education.

### Comparison with existing literature

A systematic review found adolescents (aged 11–19 years) turned to informal sources of support, such as families and friends for self-harm;^13^ this is congruent with the findings in the current study that one avenue of help-seeking young people turn to is family and friends. Parents, families, friends, and significant others, however, can be a barrier to help-seeking, and this finding builds on existing evidence that parents can have a negative impact on help-seeking in young people.^20^

In this study young people found their ‘candidacy’ as an enabler and barrier to seeking help.^25^^–^26 This study found mixed experiences of seeing GPs for self-harm and young people wanted to be understood by GPs, which is similar to the findings of Bellairs-Walsh *et al*, although their study was not solely about self-harm.^30^ The importance of the GP response and how this had an impact on the decision to seek future help was highlighted in the current study (Figure 1), corresponding to the adjudication stage of the candidacy framework — the judgements and decisions made by professionals that allow or inhibit continued progression of candidacy.^25^^–^26^,^^31^

This study found that experiences of consulting GPs for self-harm recursively affected future help-seeking from GPs, and in one instance it took one young person 3 years to seek help from a GP after a negatively perceived consultation.^32^ This has also been described in young people who are suicidal.^33^ A fear of losing confidentiality was identified as a barrier to accessing care, and this has also been reported in young people with suicidal behaviour and self-harm, and for mental health concerns in young people more widely.^30^^,^^34^^–^35 Relationship-based care, an identified facilitator for young people accessing self-harm care, is valued by patients and the Royal College of General Practitioners, and associated with improved patient satisfaction and reduced mortality.^36^^–^39 Continuity of GP care supports the building of a trusting, therapeutic relationship between GP and patient, which may help young people reduce self-harm behaviour.

### Implications for research and practice

Interviews with GPs to explore their views on managing young people who self-harm is important, and further qualitative research with young people, including those from socioeconomically deprived communities and not in higher education, using a longitudinal design will provide understanding of experiences, critical moments, and changes in self-harm over time. These findings can inform general practice-based interventions for young people who self-harm and primary care self-harm models of care, as outlined in the *NHS Long Term Plan*.^40^ As highlighted by the Samaritans, there should be the availability of evidence-informed high-intensity psychological therapies to support GP management.^41^

Young people require clear public health messages about self-harm, its risks, accessing general practice, and confidentiality. This can be done through the GP consultation, practice-level dissemination, Public Health England, local authorities, and NHS England. GPs need to adhere to current NICE guidance on self-harm management and in Box 1, the authors suggest recommendations informed by this study for GPs managing self-harm in young people. Young people want GPs who have the ability to establish and maintain relationship-based care for individuals who self-harm, and practices that are flexible when booking appointments may facilitate this.^42^^–^43

**Box 1.** Suggested recommendations for GPs managing self-harm in young people

Be aware it can be difficult for a young person to disclose self-harm behaviour, therefore, listen and act when it is disclosed. Explore and understand their self-harm: reasons, type, method, frequency, and duration, and document clearly in health records. Review medication and assess potential risk of overdose, and if starting new medication, regularly review. Discuss treatment options tailored to the needs of young people and reach shared decisions. Follow-up young people in agreement with them, even if they are waiting for a specialist assessment or psychological therapy.
